# Managing personal health information in distributed research network environments

**DOI:** 10.1186/1472-6947-13-116

**Published:** 2013-10-08

**Authors:** Christine E Bredfeldt, Amy L Butani, Roy Pardee, Paul Hitz, Sandy Padmanabhan, Gwyn Saylor

**Affiliations:** 1 Mid-Atlantic Permanente Research Institute, Kaiser Permanente Mid-Atlantic States, Rockville, MD, USA; 2 HealthPartners Institute for Education and Research, Bloomington, MN, USA; 3 Group Health Research Institute, Seattle, WA, USA; 4 Essentia Institute of Rural Health, Duluth, MN, USA; 5 C-V Sight, Shrewsbury, MA, USA; 6 Center for Health Research, Kaiser-Permanente Northwest, Portland, OR, USA

**Keywords:** HIPAA, Protected health information, Distributed research, Privacy, Security

## Abstract

**Background:**

Studying rare outcomes, new interventions and diverse populations often requires collaborations across multiple health research partners. However, transferring healthcare research data from one institution to another can increase the risk of data privacy and security breaches.

**Methods:**

A working group of multi-site research programmers evaluated the need for tools to support data security and data privacy. The group determined that data privacy support tools should: 1) allow for a range of allowable Protected Health Information (PHI); 2) clearly identify what type of data should be protected under the Health Insurance Portability and Accountability Act (HIPAA); and 3) help analysts identify which protected health information data elements are allowable in a given project and how they should be protected during data transfer. Based on these requirements we developed two performance support tools to support data programmers and site analysts in exchanging research data.

**Results:**

The first tool, a workplan template, guides the lead programmer through effectively communicating the details of multi-site programming, including how to run the program, what output the program will create, and whether the output is expected to contain protected health information. The second performance support tool is a checklist that site analysts can use to ensure that multi-site program output conforms to expectations and does not contain protected health information beyond what is allowed under the multi-site research agreements.

**Conclusions:**

Together the two tools create a formal multi-site programming workflow designed to reduce the chance of accidental PHI disclosure.

## Background

Studying rare outcomes, new interventions, and diverse populations frequently requires collaborations across multiple healthcare institutions. The ability to exchange health research data is growing through the development of distributed research networks, healthcare collaboratories, and computing grids [1-8]. As the capability for multi-site research grows, the amount of new public health research involving partnerships across academic institutions, healthcare delivery systems, insurance providers and pharmaceutical companies is also growing worldwide. For example, the HMO Research Network includes 18 independent healthcare organizations that work together on a broad range of health studies through joint participation in a virtual data warehouse [2]. Similarly, the FDA Mini-Sentinel initiative combines data from 19 collaborating institutions through a variety of distributed programming techniques [9]. In addition, the Commonwealth Government of Australia is making health data integration across institutions a high priority to support health research [10], while the United Kingdom has nationwide initiatives to support the use of electronic databases for health research across the UK [11]. These research collaborations often involve the release of de-identified patient-level information between institutions, putting patients at risk for the accidental disclosure of their protected health information (PHI). Even when only aggregate data is released between research partners, patient-level datasets are very often generated in the course of the research and can be accidentally released to collaborators. The Health Insurance Portability and Accountability Act (HIPAA) privacy rule mandates that we protect the PHI of patients in multi-site studies [12], but the specifics of how to avoid accidental disclosures in an increasingly collaborative research environment are much less clear.

This paper describes methods for protecting PHI during data exchange in collaborative research environments. Collaborative research projects generally share similar research protocols. At the beginning of the project, ethical and patient privacy review is performed by the Institutional Review Board (IRB). Approval can either be obtained at each site independently, or collaborating sites can cede IRB oversight to the lead research site. In parallel, data use agreements (DUAs) are negotiated between all involved sites. The DUA, in conjunction with the IRB agreement, details which data can be transferred between sites and how that data can be used. Once the data privacy and security documents have been approved, a number of methods can be used to accomplish the programming and data extraction, prior to data analysis. Although it is possible for some forms of data analysis to be performed at each site independently, for many projects the power and granularity of combined individual-level data is necessary [13]. In these cases, a subset of the data extracted at each site is transferred to a lead analytic site, where it is combined into a single research dataset and used for analysis. Where possible, the data transferred between institutions is de-identified through the use of study-ids and transformation or removal of other key identifiers.

The data transfer step is the most vulnerable part of collaborative research in terms of data security and data privacy. Many large research collaborations now use secure file transfer sites to reduce the likelihood of accidental disclosure outside the research environment. However, accidental disclosure of inappropriate data between members of the research team is not uncommon (personal experience and personal communications). Accidental disclosure can occur for multiple reasons: site programmers accidentally release datasets meant to be retained locally, fail to substitute study ids for patient identifiers, fail to redact small cell sizes or patients with advanced age or forget to remove identifying fields from the dataset. Problems can occur at the lead programmer’s end as well: the lead programmer can request data that is not in accord with the IRB agreement or DUA, or can accidently direct output that is meant to be retained locally to the data transfer directory. As the complexity of the project increases, the potential for all of these errors also increases.

Our goal in this work was to identify methods to reduce the potential for accidental release of PHI due to oversight. We formed a working group of HMORN site programmers and investigators (PHI Work Group) with more than 30 combined years of multi-site programming experience. We developed two approaches. The first approach is the development of an automated program to review research datasets for the presence of PHI [14]. The second approach, described in this paper, is the development of a data exchange workflow that includes a multi-site workplan template and a pre-release checklist for analysts and project managers. The tools developed for this workflow help analysts and project managers confirm that the data to be released meets the planned study release requirements.

## Methods

The PHI workgroup met regularly to 1) identify the requirements for tools to support data security and data privacy during collaborative research, and 2) develop two templates designed to support data analysts and investigators engaged in collaborative research. We focused on support of the research data analyst’s role, assuming that privacy and ethical issues have been addressed in a prior IRB approval process managed by the project investigator. We identified the following five requirements:

1. Allow for a range of PHI release characteristics. Collaborative project data can range from aggregated data containing no PHI to fully identified individual-level data. Tools designed to protect against accidental PHI disclosure should be general enough to support the entire range of allowable project data.

2. Clearly identify the common data elements protected by HIPAA for easy reference during data review.

3. Help analysts identify which PHI elements are allowed in the project based on the IRB application and approval documents supplied by the investigator. Make it easy to compare the PHI elements allowed in the IRB and DUA agreements to what the extraction program is supposed to produce.

4. Help analysts clearly identify PHI elements in the data to be released and compare them to the list of restricted PHI and to the list of PHI elements allowed in the project.

5. Prompt analysts to identify how the data is to be protected during transfer. If PHI is allowed and exists in the data to be transferred, HIPAA requires that the data be encrypted during transfer to the lead site.

Based on these requirements, we developed two performance support tools to support research analysts in conducting collaborative research.

## Results

We developed two performance support tools to guide site analysts, project managers and investigators through the process of identifying PHI in research datasets and safely releasing data to the lead research site. The first tool is a workplan template that can be customized by the lead programmer to clearly identify what site analysts should expect from the multi-site program, including what PHI the output datasets should contain. The second tool is a checklist that guides the site study team through the process of identifying PHI in the data and checking the data to be released against expectations.

### Workplan template

A comprehensive workplan ensures that site analysts have the information they need to run a multi-site program, evaluate the results, and return the results to the lead site. The workplan template guides the lead programmer through the process of writing a complete and accurate program workplan that clearly communicates program requirements to analysts at collaborating sites. Additional file 1 includes the workplan template, which can also be found at http://mapri.kaiserpermanente.org/research/mapri-sample-reports/. The workplan template has four major sections: Header, File Structure, Program-related files, Directions for running the workplan and data transfer.

The template header helps the programmer capture metadata about the project, including the project objective, timeline, contact information, the number and type of files the program generates, and a list of required input files that are distributed with the program. The completed workplan header provides a rapid overview of the program that can help site analysts manage deadlines and communicate with the lead programmer if the program doesn’t run as expected.

**Figure 1:**
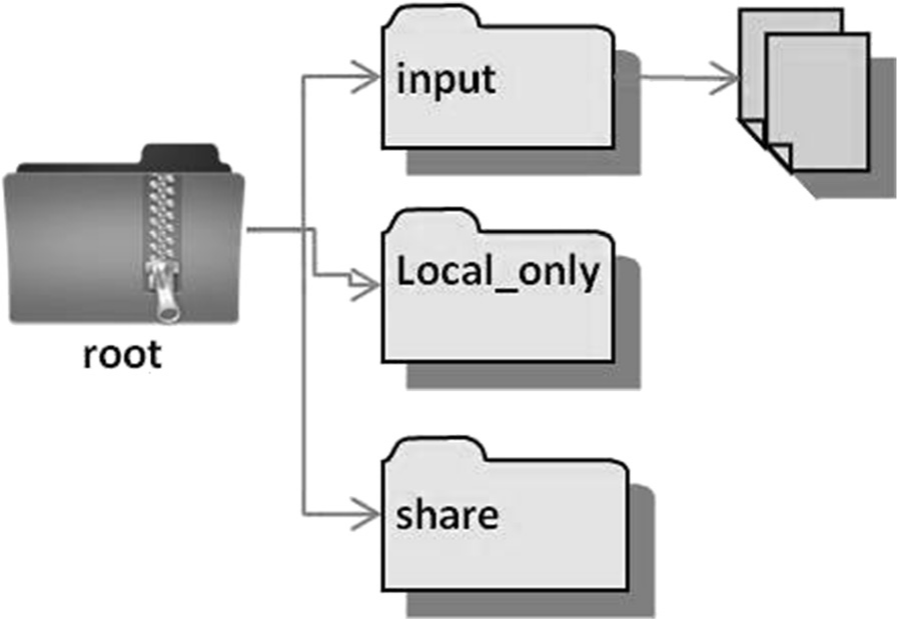
**Example of a loaded file structure.** The zip file contains the full directory structure and files needed for the multi-site program.

The second section of the workplan template leads the programmer to describe the file structure used by the multi-site program. The workplan template provides instructions to help programmers create “loaded” file structures in which all project subdirectories and files are built into a zip file. An example of a loaded zip file is shown in Figure 1. When the zip file is de-compressed by the site analyst, the only customization required is the definition of a path to the top-level project directory. Pre-creating loaded file structures reduces the chance that local and transfer files will wind up in the same directory and be transferred to the lead site together. The workplan template also encourages programmers to use two standard directory names: “share” for program output that is designed to be sent back to the lead research site, and “local only” for program output that is designed to be maintained at each research site. These file management methods are meant to encourage consistency across sites and programs, making it easier to track which files are meant to stay at the local site and which files are meant to be transferred back to the lead programming site.

The third section of the workplan template guides the programmers through describing the program input and output in order to help site analysts clearly identify which files will be used and generated by the program. In the input section, the workplan lists the local tables used by the program, as well as any study-specific input files sent with the program. In the output section, the workplan lists the files that will be generated in both the “local_only” and the “share” directories. In the “share” section, the template prompts programmers to describe any data that may be considered PHI in the datasets to be transferred back to the lead programming site, as well as any data manipulations performed to hide PHI (i.e. setting counts < 6 to 0 to prevent identification of individuals with rare diseases). The “share” section of the workplan template reminds programmers to list all the datasets meant to be transferred to the lead site, as well as any supplemental files such as logs and PDF reports.

The fourth section of the workplan template directs the lead programmer to write directions for running the multi-site program and transferring the data. The workplan directions are intended to include reminders on how to customize the program for the collaborating site and review the logs. In addition, some multi-site programs create reports that provide summary information about the datasets to help site analysts review the data. In these cases, this portion of the workplan template reminds the programmer to describe any summary reports generated by the program, point the analysts towards their location in the loaded file structure, and indicate what the analyst should look for in the report. Finally, this section of the workplan template prompts the programmer to provide instructions for returning the file to the lead research site, including whether the log files should be returned, whether the output should be compiled into a single package, whether the programmer recommends encrypting the output prior to transfer, and what transfer method should be used.

### PHI checklist

The second performance support tool consists of a checklist that the site data reviewer completes prior to releasing data. The checklist is designed to help the data reviewer confirm that project data is consistent with workplan requirements and IRB and DUA agreements. The checklist contains multiple sections that guide the data reviewer through the process of identifying the data transfer method, specifying the level of PHI that is approved for release, identifying the PHI elements in the data, and reviewing errors and other potential problems in the log file. Through the process of answering the questions on the checklist, the data reviewer is guided through the process of reviewing the data for PHI. The checklist can be found in Additional file 2, or downloaded in Word document format to allow customization for a specific site’s needs: http://mapri.kaiserpermanente.org/research/mapri-sample-reports/.

The checklist has four main sections: the data transfer method, project requirements, data review and project manager approval. The first three sections are meant to be completed by the site data analyst. The final section of the checklist provides for a secondary review by the project manager to ensure that the elements of PHI flagged in the checklist are congruent with the allowable PHI elements in the IRB agreement and DUA.

The first section of the checklist consists of prompts for the site analyst to identify where the data to be released is located, what site will be receiving the data and how it will be transferred to the lead research site. Completing this information helps ensure that the right data goes to the right place. Footnotes are used to recommend best practices for data transfer to help ensure that data is transferred in a secure method. Best practices identified in the checklist include:

• Isolating the data to be released in a single directory to make it easy to distinguish these datasets from study data that should be maintained at the collaborating site.

• Use of a secure file transfer protocol that encrypts the data during file transfer.

• Encrypting the data prior to transfer when a secure file transfer protocol is not used.

• Avoiding the use of email for file transfer due to its susceptibility to human error.

The second section of the checklist collects information about the project data. Specifically, this section requires the data reviewer to determine the level of specificity that is allowed in the transfer datasets (i.e. de-identified dataset, a limited data set, or a fully-identified dataset). If PHI is allowed in the dataset, the data reviewer is asked to specify which identifiers are allowed. In addition, the data reviewer is asked to determine whether small cell sizes (i.e. counts less than 6) are allowed in the transfer data set. The purpose of this section is to ensure that the people responsible for reviewing the data prior to transfer have a complete understanding of the types of data that are approved for release. The information used to complete this section of the checklist is drawn from the workplan, the IRB application and the DUA.

The third section of the checklist contains a list of all commonly used PHI elements. Checkboxes allow data reviewers to indicate whether the data to be transferred includes those particular elements. The data reviewer is asked to look for medical record numbers, ages over 89, birth dates, addresses, other identifiers such as accession numbers or names, and cell sizes less than 5. This section also prompts the data reviewer to review any log files being returned to the lead research site to ensure no errors or PHI are included in the log. The checklist does not try to prohibit data transfer of these items; the goal of this section is to make explicit which elements of PHI are being released from the collaborating site to permit a comparison of the existing PHI with the expected data elements based on any supporting documents such as the IRB application and the DUA.

The final section of the checklist contains instructions for project managers to do a final review on any data to be released. The checklist contains prompts for the project manager to identify where reference documents are stored, including the research protocol, the DUA, the IRB application, and the IRB approval. In addition, the project manager is prompted to verify that the characteristics of the datasets listed in the completed checklist match what was approved for release, the dataset includes the number of subjects approved for study inclusion, and that the appropriate data governing documents have been officially completed.

Both tools are available as Microsoft Word documents at http://mapri.kaiserpermanente.org/research/mapri-sample-reports/. The documents provide a starting point that can be edited to support local workflows and data security requirements.

## Discussion

An experienced group of 5 programmers and investigators developed two performance support tools to support data privacy and security in multi-site collaborate research. The first tool is a workplan template that helps programmers create consistent multi-site programs. The second tool is a PHI checklist that walks the analyst and project manager through the process of reviewing data for PHI.

Together the performance support tools meet the five requirements that were identified as necessary to support data security and privacy in a multi-site programming environment:

1. Allow for a range of PHI characteristics: Both performance support tools can be customized for projects with different levels of PHI. They both work to increase awareness of PHI elements by prompting users to consider whether PHI elements are included in the project.

2. Identify the data elements protected by HIPAA: Both the workplan template and the PHI Checklist include a listing of the most common PHI elements to remind users of the types of data that are protected by law.

3. Help analysts identify PHI elements allowed in the project: One of the goals of the workplan template is to help the lead programmer clearly communicate what PHI elements are expected to be in the program output. The PHI Checklist reminds analysts to use both the program’s workplan and the study IRB agreement and DUA to determine which PHI elements are allowable.

4. Help analysts determine whether PHI in the data is allowable. The PHI checklist contains two sections for PHI: one in which analysts are asked to determine what PHI is allowable, and a second for them to list the PHI found in the data. By comparing the two sections, analysts can determine the appropriateness of any PHI found in the data.

5. Prompt analysts to identify how data will be protected during transfer: The first section of the PHI checklist prompts analysts to identify the data transfer method, and contains information about the security of the data transfer methods.

**Figure 2:**
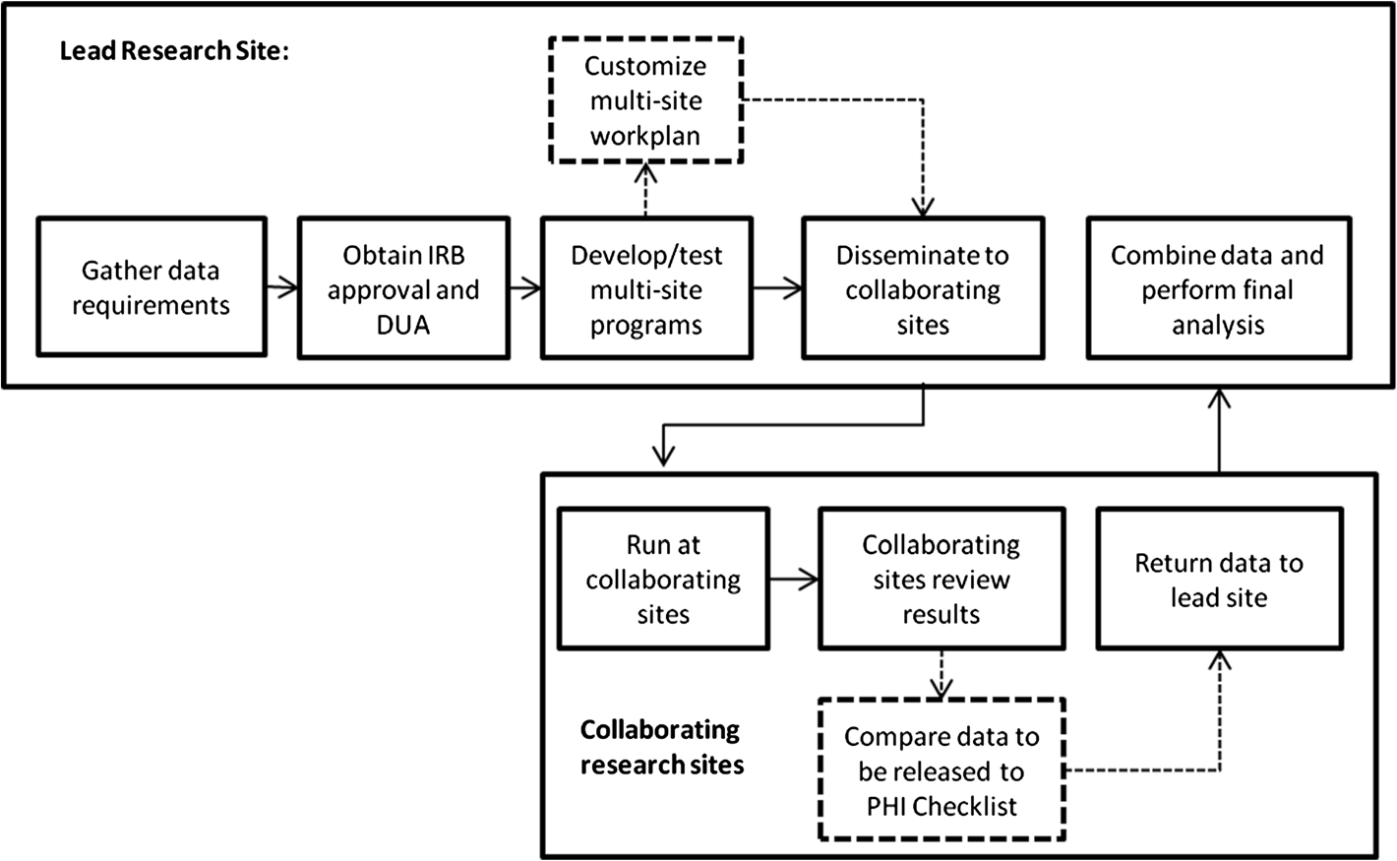
**Illustration of how the performance support tools are incorporated into the multi-site programming workflow.** Dashed lines indicate processes that are impacted by the workplan template and PHI checklist performance support tools.

The tools are designed to work together throughout the process of writing, distributing and executing multi-site research programs. Figure 2 illustrates one example of the multi-site research workflow.

The programmers work with the investigators to determine the data requirements for the study. The investigator obtains all necessary IRB approvals and DUAs which determine which data elements are allowed in the study. The lead programmer obtains a copy of the IRB application and approvals and DUA and works with the study investigators to determine the analytic plan and study data exchange requirements. The lead programmer writes the data extraction code based on common multi-site programming principles, such as those described in the HMORN toolkit (http://www.hmoresearchnetwork.org/resources/toolkit/HMORN_CollaborationToolkit.pdf). To test the data extraction program, the programmer solicits a volunteer site to run the code and evaluate the results for completeness, accuracy, and appropriateness of the data elements. The lead programmer then fills out the workplan template and creates the loaded file structure with any necessary input files. The programmer distributes the code and the completed workplan to collaborating sites. The collaborating site analysts unzip the loaded file structure, review the workplan, and customize the appropriate sections of the program. Once the collaborating site analysts have run the program, they review the data and use the program’s workplan and associated IRB and DUA documents to fill out the PHI checklist. The analyst gives the checklist to the project manager, who verifies that any PHI identified in the datasets is consistent with all relevant IRB agreements and DUAs. The analyst or project manager then creates a compressed data package including all data to be released, encrypts the data package and transfers the data to the lead site using the data transfer method identified in the first section of the PHI checklist. The lead programmer receives the data transfers from all sites, confirms the datasets are consistent with expectations, and combines the data from all collaborating sites. Throughout this process, the two performance support tools remind the programmers and analysts about best practices to help reduce the accidental disclosure of PHI.

Both of the performance support tools described here attempt to reduce accidental PHI disclosures by creating a more formal programming and review workflow. Although using workplans and checklists to create a formal workflow is not novel in the general programming environment, the combination is not as common in the multi-site health research programming community. Given the negative consequences associated with accidental release of PHI, applying these techniques will reduce the data security risks of multi-site collaborations, while also making multi-site projects more efficient through standardization of the review process.

The goal of the methods presented here is to improve communication between the lead programmer and site analysts, and to increase awareness of PHI elements in datasets that are being transferred back to the lead research site. An additional method to improve data privacy and security is to use programmatic techniques to summarize the data for easy review. For example, when the multi-site program finishes processing the data, it could create a report indicating which output files were created, whether the output contained fieldnames indicating key elements of PHI such as “Medical_Record_Number”, and whether the numerical output contained values that might indicate small populations. In addition, if a research project were limited to females between the ages of 18 and 55, the report could contain gender and age ranges for the population in the dataset to make it easier for the data reviewer to confirm that the data contains the appropriate population. This type of report would make it easier for the data reviewer to evaluate the data, as it’s difficult to check every record in a large, complex dataset by hand. The data in the report could be used to help complete the PHI checklist, and could also be sent back to the lead site to enable quick review that the data conforms to expectations.

The information tools and workflows presented here form part of a larger information governance framework that supports the activities of the HMO Research Network (HMORN). The HMORN’s information governance framework includes multiple processes designed to increase the efficiency and security of multi-site research (http://www.hmoresearchnetwork.org/resources/toolkit/HMORN_CollaborationToolkit.pdf). Process improvements include standardized, pre-negotiated data use agreements and subcontracting templates that all HMORN members agree to use, facilitated IRB review across sites, and guides for using the shared data structures (Virtual Data Warehouse). Additional guides for project policy, recruitment, interviews and multi-site chart abstraction are all available to help multisite research personnel collaborate effectively and efficiently. The workplan template and PHI checklist described above support the overall HMORN workflow by providing consistent workflows for the multi-site programming approach. By following these workflows, projects decrease the probability of accidental PHI release and improve the communication between programmers at disparate sites.

## Conclusions

We developed two performance support tools to help health research programmers protect both data privacy and data security in multi-site, collaborative projects. The workplan template guides the lead programmer through creating a comprehensive workplan that will support site analysts in running and evaluating the multi-site program. The PHI checklist guides the site analyst through reviewing the program data to identify PHI elements and determine whether the PHI is allowable under project agreements. Together, the two performance support tools create a more formalized programming environment that encourages effective communication and data review.

## Availability and requirements

Both performance support tools are attached to this manuscript for review. On publication, the tools will be made available on the Mid-Atlantic Permanente Research Institute’s website.

## Abbreviations

PHI: Protected health information; HIPAA: Health insurance portability and accountability act.

## Competing interests

The authors declare they have no competing interests.

## Authors’ contributions

CB drafted the design of the PHI Checklist and drafted the manuscript. AB contributed to the content of both tools, finalized design of PHI Checklist and organized regular study meetings. RP contributed to the content and design of both performance tools and provided technical assistance. PH contributed to content and design of both performance tools. SP drafted the content of the PHI checklist and organized regular study meetings. GS drafted the content and design of the workplan template. All authors read and approved the final manuscript.

## Pre-publication history

The pre-publication history for this paper can be accessed here:

http://www.biomedcentral.com/1472-6947/13/116/prepub

## Supplementary Material

Additional file 1 Workplan template for communicating program requirements. Click here for file

Additional file 2 Data release checklist for study teams. Click here for file
