# Supplementary material for: Managing personal health information in distributed research network environments
Source: BMC Med Inform Decis Mak. 2013 Oct 8;13:116. doi: 10.1186/1472-6947-13-116 (PMC3851487; doi:10.1186/1472-6947-13-116)
Supplement: Additional file 1 — Workplan template for communicating program requirements. [file 1472-6947-13-116-S1.doc]

| 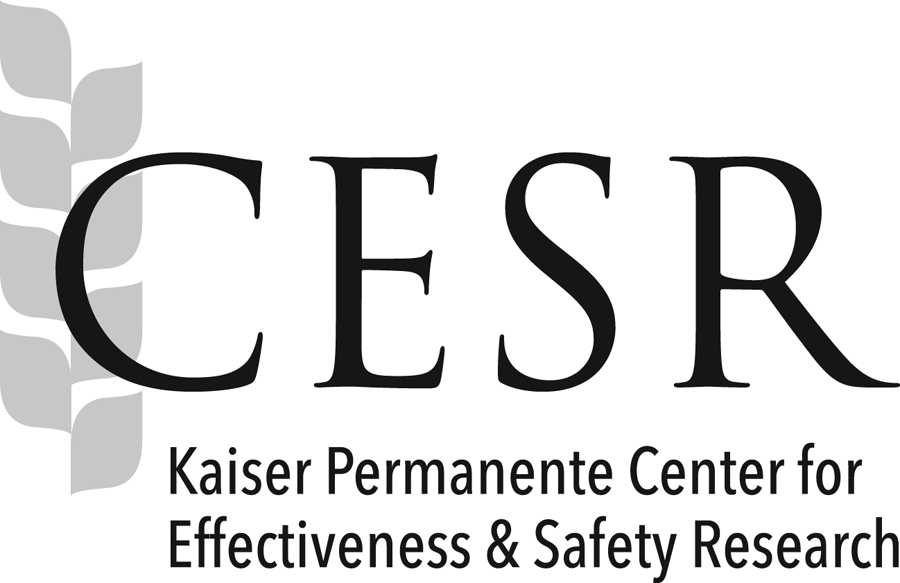 |
| --- |
| Multi-Site Workplan Template |
| Directions and Explanations Included |
|  |
| **Compiled by Gwyn Saylor**  **Senior Research Network Consultant**  **CESR Data Coordinating Center** |
| **September 2013**  **Every VDW programmer who has written or run a multi-site program**  **has contributed in some way to this document.** |

| The following is only a starting place and should be adapted and adjusted as needed by each project. All blue in the workplan header should be overtyped and filled in by the programmer developing the workplan. In the body of the workplan yellow is used to highlight directions to the developer and should not appear in the final workplan.  Hopefully, using this template will make it easier and quicker for the developing programmer to create a workplan that communicates clearly, concisely and completely. Using this template will assist programmers who will be running an accompanying program by creating a consistent workplan layout. Familiarity will develop. Programmers will know where to look for a particular piece of information, what to expect in program output, and what specific folder contains files that should be returned to the lead site.  Much care and thought is necessary to write a workplan that anticipates and answers all of a user’s questions. Read through and tweak your workplan several times. Try to put yourself in the place of someone who isn’t familiar with the project at all and is only funded to run a plug and play. |
| --- |

study_short_name_wp0xv0y (workplan 0x version 0y)

Date submitted: Month dd, yyyy

| **Project Name:** Full Study Name | | | |
| --- | --- | --- | --- |
| **Overall Project Objective:** a short paragraph giving the programmer a general idea about the goals of the study | | | |
| **Requesting PI Contact Information** | | **Requesting Programmer Contact Information** | |
| Name: |  | Name: |  |
| Institution: |  | Institution: |  |
| Phone #: |  | Phone #: |  |
| Email: | create email link | Email: | create email link |
|  | | | |
| **Other PIs:** | | | |
| **Project Stage (feasibility, pilot data, funded, …):** | | | |
| **Workplan Timeline:** Please return requested files by mm/dd/yy | | | |
| **Workplan Package Zip File:** name of the zip file. (tip: we suggest study_short_name_wp0xv0y.zip)  **Files Included in Zip File:**  sas\study_short_name_wp0xv0y.sas  input\ (list SAS data sets or any other file to be used as input)  document\ study_short_name_wp0xv0y.doc and any other support documentation sent with the workplan | | | |
| **Number and Type of Files to be Returned:** number of SAS data sets, number of logs, number of PDFs, … | | | |
| **Workplan purpose:** a short paragraph describing what this program does, file specifics will be listed below | | | |

Storage:

The file structure is determined by the lead site and a ‘loaded’ copy of this structure is sent with the workplan. ‘Loaded’ means that all files supplied by the lead site and pertinent to the completion of the workplan are located in the appropriate folder within the structure. The structure is zipped and passed to the participating sites via a secure transfer website. Upon unzipping the package at the root location, the ‘loaded’ structure opens. The root folder location is determined by each site. The folder names listed below the root follow a general pattern determined by the VDW Implementation Group (VIG), but most of the names and number of folders can be altered to meet a study’s needs. Two folders will have the same name and use across all studies to promote PHI safety. All files to be shared outside of a site and only files to be shared outside a site will be written to a folder called share. All files to be retained at the participating site will be written to a folder called local_only.

root – Please determine the location and name of this workplans’ root folder. For the folder name consider appending the date onto the workplan name 🡺 study_short_name_wp0xv0y_yyyymmdd. After expanding the zip file into the root folder you will see the following 5 folders.

- document – This folder contains any descriptive information about the project. The workplan is stored in this folder.
- input – This folder contains any files used by the program and supplied by the lead site.
- local_only – This folder contains all files generated by the program that are to be retained by the participating site. This folder can be partitioned with sub-folders if desired by the lead site.
- sas – This folder is where SAS code is stored. Log and Lst files can also be stored here.
- share – This folder contains all reports, SAS data sets, and any other files to be shared outside the participating site. Logs could be stored here if they are to be returned to the lead site. This folder can be partitioned with sub-folders if desired by the lead site.

A note about case: Folder names should be lowercase. Many different operating systems are used across the various sites. Lowercase was preferred most often by sites where case makes a difference. Any letters in the names of files contained in these folders should be lowercase. All path names, folders and file names (other than SAS datasets) referenced in SAS code should be lowercase.

input – description/listing of files used by the sas program:

- **Supplemental files sent with workplan:**
  - Explain how and why each supplemental file sent with the workplan will be used.
- **Other input files that may not be stored under the root directory:**
- **Existing Files or Programs already at your site:**
- stdvars.sas
- If a file that was created by earlier programs or sent to the participating sites at an earlier time will be accessed explain why and how.
- Local VDW data sets:
  - List the VDW content areas and what years of data will be accessed.

local_only – output files to be kept at your site:

- List all files that will be stored in local-only or in its sub-folders.
- review_wp0xv0y.pdf report is produced in the final portion of the workplan’s program and is stored in local_only. The report contains:
  - PROC CONTENTS and PROC PRINT of each SAS file in share and its sub-folders
  - A listing of variables found in SAS files in the share folder that are deemed sensitive or to possibly contain PHI.
  - Crosstabs and summary tables to assist the programmer in confirming that data to be returned contains only what it is supposed to contain. ( Such as: females, age 21 to 65, 2004-2012,…) Each report should be tied to the relevant data set by titles and footnotes.

share – output files to be sent via secure file transfer website to lead site:

- List each file that will be stored in share or in its sub-folders.
  - Explain what a file contains and what each record represents
  - State if PHI is contained or not
  - State if counts contain values less than &lowest_count. (&lowest_count is a macro variable created by stdvars.sas. Each site can establish the value of &lowest_count to serve as the least number of records that will be counted.

**Running this workplan:**

- Create a folder on your site’s local file service that will contain the content for this request. Download the zip file. Unzip it into the folder you created. The subfolders will automatically be created. The folder structure will look like the following with the name of the “root” directory specified by you.

- The workplan program has a clearly marked edit section near the top of the program. Please complete the edits as directed by comments and examples near the top of the program.
- Review the logs (one in the **sas** folder and one in the **share** folder) for errors and warnings. If there are problems and you send a log to the lead site make sure the log is redacted of PHI and any site specific information that your site does not want released.
- After the program runs successfully and before releasing any results please review the review_wp0xv0y.pdf file found in the **local_only** folder. This report is to assist the programmer at each site in determining that only information that conforms to the data use agreement in place is being shared with the lead site.
  - This file contains PROC CONTENTS and PROC PRINT output for each data set that was written to the share folder. It also utilizes the Detect_PHI macro to list variables found in SAS datasets in the share folder that are deemed to be sensitive or possibly contain PHI.
  - Review this output and any other summary tables or output created by crosstabs.
  - Confirm that the files conform to the data use agreements in place.

Directions to transfer data:

- Please zip all files in the SHARE folder into one zip file called:

Give directions how to name the zip file. A suggestion:  **study_short_name_wp0xv0y_&_siteabbr._yyyymmdd.**

- Upload zip file to the create a link to the secure file transfer site being used.
- Place the zip file in the study folder called fill in the name.
- Once the file has been uploaded to the transfer site please let me know that your region has completed this workplan by emailing fill in your email address.
