# Supplementary material for: Managing personal health information in distributed research network environments
Source: BMC Med Inform Decis Mak. 2013 Oct 8;13:116. doi: 10.1186/1472-6947-13-116 (PMC3851487; doi:10.1186/1472-6947-13-116)
Supplement: Additional file 2 — Data release checklist for study teams. [file 1472-6947-13-116-S2.pdf]

Print Form

Data Release Checklist for Study Teams

Abbreviated Title:	Last Name of PI:	IRB#
Name of programmer:	Programmer phone number:	

Instructions For Programmers:

Please fill out the below and give this checklist and any associated documents to the project manager.

Directory containing the data to be released[1]: ________________________________

Receiving Site: __________________________________

**Means of Transfer**	
○ **Secure Transfer Website**	**URL:**
○ **E-mail Attachment[2]**	**Recipient:**
○ **SFTP**	**Server:**
○ **FTP[3]**	
**SSH**	
○ **Mini-Sentinel Query Tool Client**	**Requester:**
○ **PopMedNet Query Tool Client**	
○ **SPAN Query Tool Client**	
○ **Postal Mail[4]**	**Address:**
**Other method**	**Please specify:**

Comments on Means of Transfer:

____

____

____

Will the data files be encrypted prior to transfer?

○ **Yes**

○ **No**

---

[1] The PHI workgroup recommends isolating just the data to be sent in a single directory, to make it easy to distinguish these datasets from any other study data that should not be sent.

[2] The PHI workgroup recommends against use of e-mail for transferring PHI-laden study data, because of how easy it is to mistype the recipient's e-mail address, and how easy it is for the data to be inadvertently forwarded by the recipient. Any PHI sent via e-mail attachments should be strongly encrypted, and any passwords should be sent via a different channel (e.g., a telephone call).

[3] Because FTP transmissions are not encrypted as part of the protocol, any PHI-laden data sent via FTP should be strongly encrypted.

[4] Here too, any PHI-laden data sent via postal mail should be strongly encrypted, and any passwords should be sent via a different channel (e.g., a e-mail).

Time period of data requested: Start date: ____________End date: ________________

Type of data that are approved for release

Aggregate (count) data

- ○ cells < 5 masked
- ○ cells < 5 NOT masked

Individual Person or Event-Level Data

- ○ Deidentified
- ○ limited dataset: dates, please specify:____
- ○ limited dataset: geographic information such as census tract
- ○ specific identifiers, please specify:____
- ○ last names with no additional information (e.g., to be run through the GUESS program)

Other Data

___Other—please specify:____

Sources of data included in release:

	Yes	No	From Date	To Date
Enrollment	☐	☐		
Demographics	☐	☐		
Encounters	☐	☐		
Diagnoses	☐	☐		
Procedures	☐	☐		
Pharmacy	☐	☐		
Lab Results	☐	☐		
Provider	☐	☐		
Death	☐	☐		
Vitals	☐	☐		
Census	☐	☐		
Site-specific data sources (specify):	☐	☐		

Does the data to be transferred include any of the following?

Yes	No	N/A	
☐	☐	☐	Medical Record Numbers (MRNs)
☐	☐	☐	StudyIDs—project-specific person identifiers
☐	☐	☐	Ages over 89
☐	☐	☐	Birth date
☐	☐	☐	Address or city
☐	☐	☐	Zip codes
☐	☐	☐	Other IDs, such as accession number, medical history number
☐	☐	☐	Cell sizes <5 (Can you group cells or send less data?)
☐	☐	☐	Files meant to be retained locally are not in the study output directory

Have you inspected a Proc Contents listing (or equivalent) for each dataset to be transferred and attached a copy to this checklist for Project Manager inspection/approval?
☐ Yes ☐ No ☐ N/A

Log is being returned to the coordinating site:
☐ Yes ☐ No ☐ N/A

If Log is requested, please check the following:
☐ Yes ☐ No Does the log have errors that display data?
☐ Yes ☐ No Does the log have warnings that display data?
☐ Yes ☐ No Does the log have notes that display data?
☐ Yes ☐ No Have you deleted any such data displayed in the log file?
☐ Yes ☐ No Does the macro option display resolved values that contain PHI?
☐ Yes ☐ No Log has been redacted for small cell counts.

Signed:________________________________ Date:_________________
Programmer

Instructions for project managers:

Location of reference documents

Protocol: ___________________________________
MOU/DUA: ___________________________________
IRB Application: _____________________________
IRB Approval: _______________________________

Please compare the release characteristics on the data checklist above to the IRB application and MOU/DUA.

DONE

☐ 1) Verify that the data set as described above is what was approved for Release (and is covered by the DUA or other contract if needed)
☐ 2) Verify that this data set includes only the number of subjects that approved for inclusion in the study.
☐ 3) Verify that the appropriate agreements for this release are in place (i.e., data use agreement, memorandum of understanding, contract, consent form, etc).

Signed:________________________________ Date:_________________
Project Manager
